# Supplementary material for: Adoption and continued use of mobile contact tracing technology: multilevel explanations from a three-wave panel survey and linked data
Source: BMJ Open. 2022 Jan 17;12(1):e053327. doi: 10.1136/bmjopen-2021-053327 (PMC8764714; doi:10.1136/bmjopen-2021-053327)
Supplement: Supplementary data [file bmjopen-2021-053327supp001.pdf]

Appendix: Adoption and continued use of mobile contact tracing technology

Figure A1: Missing data patterns across attitudinal predictors + adoption (DV, wave 2)

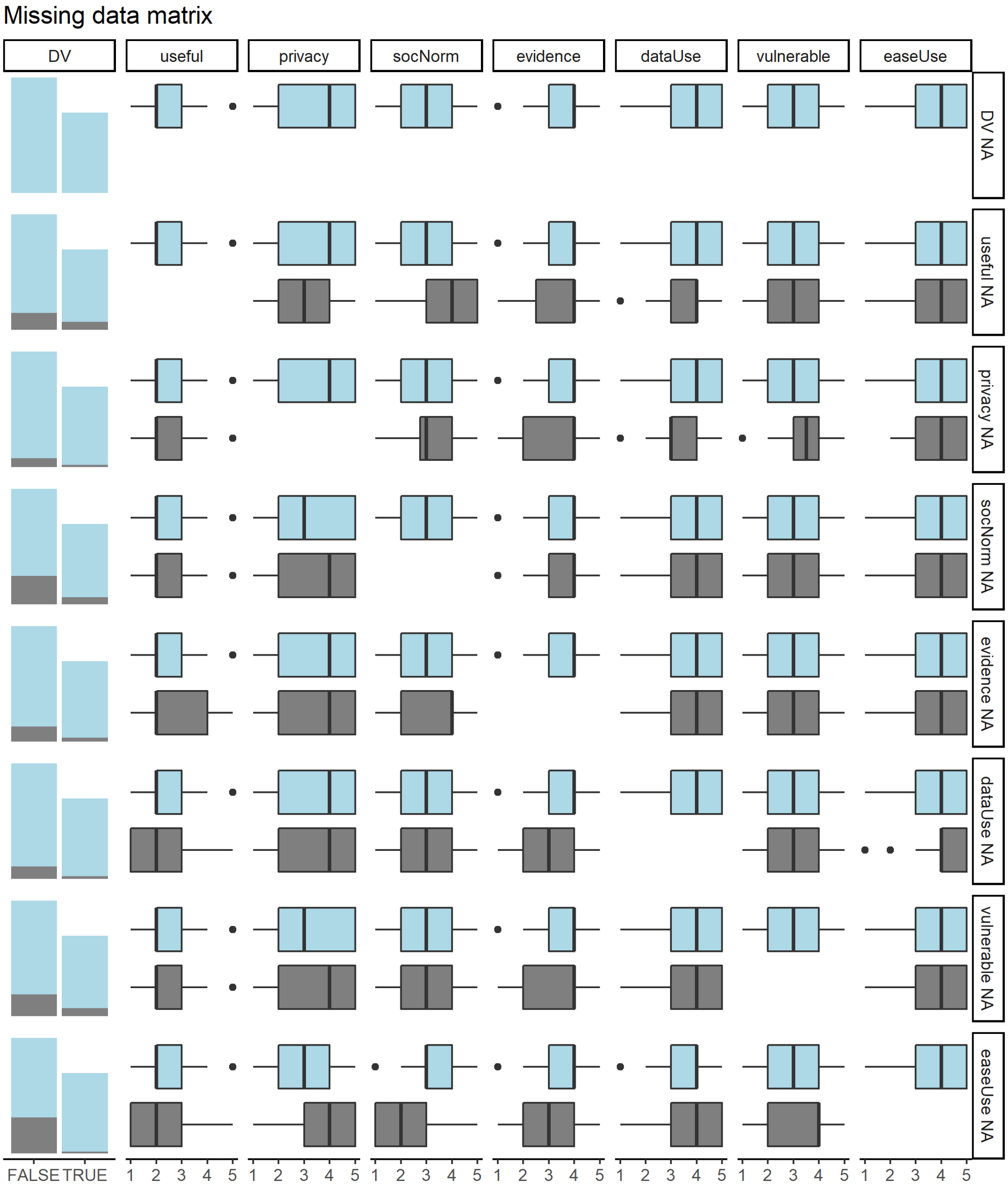

Notes: attitudinal predictors more likely to be missing for non-adopters, particularly "ease of use"; some but not all attitudes are related to each other in terms of missingness, for example those with missing data on ease of use were more concerned about privacy.

Horvath L, *et al. BMJ Open* 2022; 12:e053327. doi: 10.1136/bmjopen-2021-053327

Table A1: Logistic regression results pooled across five multiply imputed*** datasets

Model	Term	Log odds	OR	SE	p	RIV*	Lambda**
	Intercept	-0.82	0.44	0.23	0.00	0.02	0.02
	Age	-0.18	0.84	0.06	0.00	0.04	0.04
	Women	0.01	1.01	0.10	0.89	0.01	0.01
	Education	0.00	1.00	0.11	0.97	0.06	0.06
	Ethnic minorities	-0.03	0.97	0.19	0.87	0.04	0.04
**Initial adoption**	Perceived usefulness	-0.03	0.97	0.06	0.61	0.42	0.30
N = 2,500	Privacy concern	-0.81	0.44	0.07	0.00	0.05	0.05
N (districts) = 1,282	Social norms	0.60	1.82	0.06	0.00	0.56	0.36
SD (districts) = 0.34	Transparent evidence	0.13	1.13	0.06	0.05	0.14	0.12
m = 5***	More info on data usage	0.12	1.12	0.07	0.13	0.19	0.16
	Evidence on vulnerable groups	0.01	1.01	0.06	0.89	0.15	0.13
AIC = 2596.07	Ease of use	0.61	1.84	0.06	0.00	0.14	0.12
BIC = 2712.55	Trust in UK Gov't	-0.04	0.96	0.05	0.43	0.04	0.04
logLik = -1278.03	Mobility	-0.20	0.82	0.11	0.08	0.04	0.04
df.resid = 2,480	Compliance	0.29	1.33	0.12	0.02	0.04	0.04
	Tier 2 (district)	-0.19	0.83	0.11	0.10	0.01	0.01
	Tier 3 (district)	-0.20	0.82	0.16	0.22	0.03	0.03
	Cases (district)	0.03	1.03	0.05	0.60	0.07	0.07
	Urban (district)	0.33	1.39	0.18	0.08	0.01	0.01
	Intercept	1.67	5.29	0.28	0.00	0.01	0.01
	Age	0.12	1.13	0.11	0.28	0.01	0.01
	Women	0.17	1.19	0.21	0.41	0.01	0.01
	Education	0.28	1.32	0.21	0.19	0.01	0.01
**Continued use**	Ethnic minorities	-0.28	0.76	0.35	0.43	0.00	0.00
N = 1,026	Perceived usefulness	0.16	1.18	0.11	0.16	0.10	0.09
m = 5***	Privacy concern	-0.26	0.77	0.13	0.05	0.03	0.03
	Social norms	0.27	1.31	0.13	0.06	0.27	0.21
AIC = 742.12	Transparent evidence	-0.25	0.78	0.14	0.08	0.09	0.08
BIC = 816.12	More info on data usage	0.00	1.00	0.14	1.00	0.10	0.09
logLik = -356.06	Evidence on vulnerable groups	-0.03	0.97	0.12	0.84	0.12	0.11
df.resid = 1,011	Ease of use	0.20	1.22	0.13	0.13	0.03	0.03
	Trust in UK Gov't	-0.04	0.96	0.11	0.69	0.01	0.01
	Mobility	0.06	1.06	0.22	0.78	0.00	0.00
	Compliance	-0.16	0.85	0.22	0.45	0.01	0.01
	Intercept	-3.31	0.04	0.32	0.00	0.02	0.01
	Age	-0.49	0.62	0.12	0.00	0.02	0.01
	Women	0.29	1.34	0.22	0.19	0.00	0.00
	Education	0.52	1.68	0.23	0.02	0.01	0.01
**New adoption**	Ethnic minorities	-0.22	0.80	0.38	0.56	0.01	0.01
N = 1,474	Perceived usefulness	0.04	1.04	0.12	0.77	0.07	0.07
m = 5***	Privacy concern	-0.37	0.69	0.15	0.02	0.02	0.02
	Social norms	0.34	1.40	0.13	0.02	0.16	0.14
AIC = 688.88	Transparent evidence	0.18	1.20	0.14	0.19	0.08	0.08
BIC = 768.32	More info on data usage	0.06	1.07	0.15	0.69	0.12	0.11
logLik = -329.44	Evidence on vulnerable groups	0.08	1.08	0.13	0.55	0.05	0.05
df.resid = 1,459	Ease of use	-0.04	0.96	0.13	0.77	0.31	0.24
	Trust in UK Gov't	0.34	1.40	0.12	0.01	0.01	0.01
	Mobility	0.29	1.34	0.25	0.25	0.00	0.00
	Compliance	0.11	1.12	0.23	0.63	0.00	0.00

* Relative increase in variance due to missingness
** Proportion of total variance due to missingness
*** Number of multiple imputations generated with Gibbs sampling (predictive means) implemented in `mice` (Van Buuren and Groothuis-Oudshoorn, 2011).

Horvath L, *et al. BMJ Open* 2022; 12:e053327. doi: 10.1136/bmjopen-2021-053327
